# A Mathematical Model to Study the Mechanical Information Induced by Lifting-Thrusting Needle

**DOI:** 10.1155/2019/5475426

**Published:** 2019-04-08

**Authors:** Yi Yu, Wei Yao, Guanghong Ding

**Affiliations:** Department of Aeronautics and Astronautics, Fudan University, Shanghai Research Center of Acupuncture, 220 Handan Road, Shanghai, 200433, China

## Abstract

Focusing on the mechanical effect of traditional Chinese acupuncture, this study builds a mathematical model that simulates the mechanical process of lifting-thrusting needle. Analytic and numerical solutions are obtained to explore the mechanical information (displacement, strain, stress, and energy) in the skin tissue. Our results show that (1) needle manipulation leads to tissue displacement and mechanical stress field, but the needle should be inserted into the right position (about *π*/*ω* cm around the acupoint, where *ω* is the angular frequency) and enough depth (about 2 cm in lower limbs) to achieve effective mechanical stimuli; (2) the tissue displacement decays with an increase of distance from the stimulus position, more rapidly at higher frequencies; (3) there is an inverse relationship between the area of the ‘effective influence region' (where shear strain > 0.2) and the stimulus frequency, which means larger needle movement is needed at higher frequencies to achieve a better curative effect; (4) more energy is required to maintain high frequency manipulation. This study proposes a preliminary comprehension of the mechanical response around the needle during the acupuncture process.

## 1. Introduction

Acupuncture, a physiotherapy sourced from Traditional Chinese Medicine (TCM), now gains recognition all around the world. Its therapeutic effect has been widely accepted since the National Institutes of Health Hearing in 1997 [1]. Acupuncture is a biomechanical process, during which a needle is inserted into the skin at the acupoint, and mechanical stimulation to the tissue is generated when twisting or lifting the needle. Elucidating the mechanical and biological mechanism of acupuncture effect is one of the urgent problems in TCM as well as international researches on physiotherapy.

The premise to accomplish acupuncture effect is to get sensations (“De Qi” in Chinese), which means to maintain a certain intensity of stimulation by manipulating the needle. However, “De Qi” is usually described subjectively according to the experiences of skillful acupuncturists and the feelings of some sensitive patients; the underlying issues are lack of quantitative research. Therefore, it is important to make clear the mechanical sensation in acupuncture and to reveal the key factors related to it.

**Figure 1:**
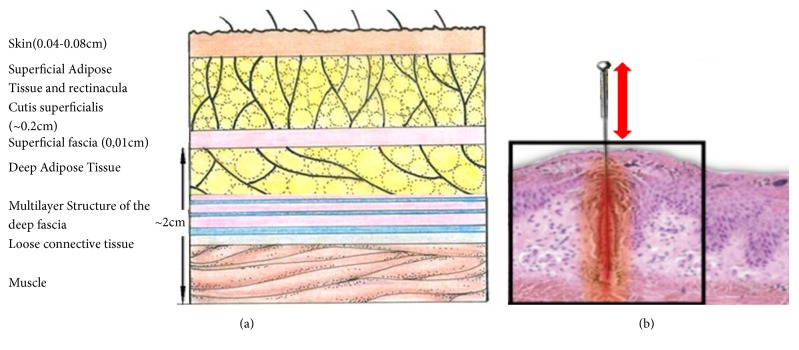
Acupoint morphology. (a) Basic pattern of organization of subcutaneous layers [9, 10]; (b) acupuncture location in lower limbs.

Numerous work and different explanations have been put forward to help understand the acupuncture process. Recent morphological studies have made clear that the acupoint is mainly composed of connective tissue. As shown in Figure 1, the dermic dense connective tissue and subcutaneous loose connective tissue form a three-dimensional collagen fiber network with numerous intertwining blood vessels, nerves, mast cells, and lymphatic vessels [2, 3]. Goh et al. hypothesized that there might be a relationship between the efficacy of acupuncture treatment and the depth of insertion needle [4]. Langevin et al. hypothesized that needle manipulation generates mechanical signals at acupoints [5], where longitudinal waves and transverse waves can propagate. Acupuncture needle manipulation results in sustained stretching by lifting-thrusting or rotating in the tissue of the acupoint [6]. Yu et al.'s experiment showed that the acupuncture effect and the force on the needle are very significant when the collagen structure is complete, and when the collagen is destroyed, the acupuncture effect is significantly weakened and the force on the needle body is very small [7]. Mvogo et al. constructed the analytic solutions of the tissue with collagen [8].

The mechanical effect of needle insertion and manipulation is the key to acupuncture; however, researches related with the quantitative mechanical effect of acupuncture are limited. Deleuze et al. has numerically simulated the features of subcutaneous interstitial flow induced by acupuncture [11]. Thiriet et al.'s mathematical model analyzed the different effects between acupuncture positioned at and out of acupoints [12]. We have also constructed a mathematical model to analyze the needle rotation at acupoints [13]. In this study, the previous work is extended, and the acupuncture of lifting-thrusting manipulation is studied through the establishment of a mechanical model based on the theory of viscoelastic mechanics. The influences of needle factors, such as stimulus frequency, movement amplitude, radius, and insertion depth, have been discussed in order to explore the acupuncture mechanical effect.

## 2. Methods

### 2.1. Model

**Figure 2:**
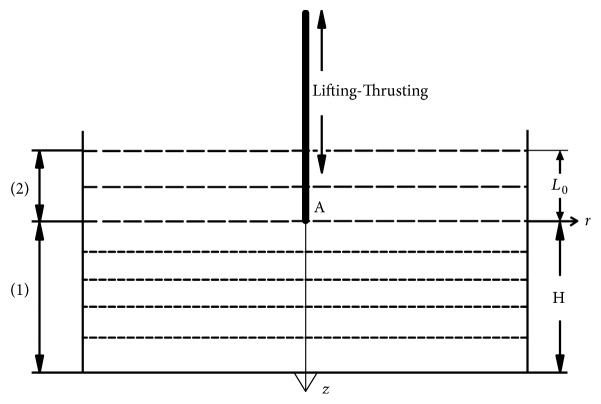
Diagrammatic sketch of lifting-thrusting manipulation.

Soft tissues are usually assumed as viscoelastic materials; some are more “elastic” and some are more “viscous”; they exhibit obvious hysteresis, stress relaxation, and other viscoelastic phenomena [14]. In our model, the skin tissue is treated as a semi-infinite viscoelastic body and the needle is treated as a rigid body. As shown in Figure 2, the tissue is divided into two parts. Part (1) represents the tissue from the plane of the tip location to infinity (*z* ≥0) where tissue displacement is caused by the movement of the needle tip (point A). Part (2) is the needle manipulation region (– *L*_0_≤ *z* < 0), where *L*_0_ is the needle insertion depth and the standard *L*_0_ is 2 cm in lower limbs.

### 2.2. Equations

The soft tissue is supposed as a homogeneous isotropic incompressible viscoelastic body and fit for Feng's quasilinear viscoelastic theory of small amplitude displacement [15]. For acupuncture manipulation, the needle movement causes small amplitude oscillatory displacements in the low stress range (physiological state) of the tissue. The boundary conditions are specified as simple harmonic functions of time; therefore, the variables can be expressed as

(1) uixi,t=u~ieiωtσijxi,t=σ~ijeiωtεijxi,t=ε~ijeiωti,j=1,2,3.

where *u*_*i*_ is the displacement component which is the distance from the original (equilibrium) position, *x*_*i*_ is the coordinate component, *t* is the time, *ω* is the angular frequency, *σ*_*ij*_ is the component of stress tensor, and *ε*_*ij*_ is the component of strain tensor.

Based on the strain-displacement relation and excluding the effect of volume force, we get the governing equation of motion in linear viscoelastic medium

(2) $$\begin{array}{c} \left[\;\lambda^{*}\left(\;i\omega \;\right)+\mu^{*}\left(\;i\omega \;\right)\;\right]du_{j,ji}+\mu^{*}\left(\;i\omega \;\right)du_{i,jj}=\rho \frac{\partial^{2}u_{i}}{\partial t^{2}}. \end{array}$$

where *μ*^*∗*^(*iω*) and *λ*^*∗*^(*iω*) are the viscoelastic material parameters and* ρ* is the density. Postulate the tissue is incompressible, that is, *u*_*j*,*ji*_ = 0, and putting (1) into (2), we get

(3) $$\begin{array}{c} \mu^{*}\left(\;i\omega \;\right)d{\tilde{u}}_{i,jj}=-\rho \omega^{2}{\tilde{u}}_{i} \end{array}$$

Rewrite it in tensor form

(4) $$\begin{array}{c} \mu^{*}\left(\;i\omega \;\right)\nabla^{2}\vec{\tilde{u}}=-\rho \omega^{2}\vec{\tilde{u}} \end{array}$$

Suppose the tissue around the needle is a semi-infinite space. This is an axisymmetric problem (*u*_*θ*_ = 0,∂/∂*θ* = 0). Replace *μ*^*∗*^ with *μ* and $\tilde{u}$ with *u*; then we have (5) in cylindrical coordinate

(5) μ∂2ur∂r2+1r∂ur∂r−urr2+∂2ur∂z2=−ρω2urμ∂2uz∂r2+1r∂uz∂r+∂2uz∂z2=−ρω2uz.

where *u*_*r*_ and *u*_*z*_ are the displacement amplitude in* r* and* z* direction, respectively. Defining the dimensionless parameters: *U*_*Z*_ = *u*_*z*_/*u*_0_, *U*_*R*_ = *u*_*r*_/*u*_0_, *Z* = *z*/*u*_0_, *R* = *r*/*u*_0_, where *u*_0_ is the amplitude of needle movement. Then (5) is changed to

(6a) μ∂2UR∂R2+1R∂UR∂R−URR2+∂2UR∂Z2=−ρω2u02UR

(6b) μ∂2UZ∂R2+1R∂UZ∂R+∂2UZ∂Z2=−ρω2u02UZ.

Based on the strain-displacement relation

(7) εrz12∂uz∂r+∂ur∂zeiωt=12∂UZ∂R+∂UR∂Zeiωt=ERZeiωt.

where *E*_*RZ*_ is calculated by explicit difference scheme

(8) ERZ12∂UZ∂R+∂UR∂Z≈12ΔRUZj+1,k−UZj,k+12ΔZURj,k+1−URj,k.

And the corresponding stress is

(9) τrzRe2μiωεrz=Reμ1+iμ2·2ERZcos⁡ωt+i sinωt=2ERZμ1cos⁡ωt−μ2sin⁡ωt=2ERZμcos⁡ωt+δ.

where $\left|\mu \right|=\sqrt{\mu_{1}^{2}+\mu_{2}^{2}}$, tan⁡*δ* = *μ*_2_/*μ*_1_, and *δ* is the phase difference between stress and strain. The shear stress amplitude *T*_*RZ*_ = 2|*μ*|*E*_*RZ*_.

In a cycle, the energy dissipation of one-unit volume caused by lifting-thrusting is

(10) ΔE=∫02π/ωτrzε˙rzdt=4μ·ERZ2∫02π/ω−ωμ1cos⁡ωt−μ2sin⁡ωt·sin⁡ωtdt=4πERZ2μ2.

The energy dissipation of the whole domain is

(11) Ef∫r0/u0+∞∫−L0/u0+∞ΔEdZdR=∫r0/u0+∞∫−L0/u0+∞4πμ2ERZ2dZdR.

Define *E*_max_ as the max energy stored

(12) Emax4πERZ2∫0π/ωμ1ωcos⁡ωtsin⁡ωtdt=2πμ1ERZ2.

Therefore, the mechanical quality factor* Q* which reflect the ratio of energy storage to energy dissipation is

(13) $$\begin{array}{c} Q=\frac{2\pi E_{max}}{\Delta E}=\frac{\mu_{1}}{\mu_{2}}. \end{array}$$

### 2.3. Boundary Conditions

The motion of the boundary is

(14) ur=0,uz=u0cos⁡ωtr=r0,  −L0≤z≤0ur=uz=0z→∞uz=0r→∞,

where* r*_0_ is the radius of the needle. Then

(15) UR=0R→0∪R→∞∪Z→∞UZ=0R→∞∪Z→∞UZ=1R→0∩−L0u0≤Z≤0.

### 2.4. Analytic Solutions of Part (1)

Let *U*_*R*_ = *F*(*R*)*G*(*Z*); (6a) is expressed as

(16) $$\begin{array}{c} \frac{F_{RR}+\left(1/R\right)F_{R}-\left(1/R^{2}\right)F}{F}+\frac{G_{ZZ}}{G}+\frac{\rho \omega^{2}{u_{0}}^{2}}{\mu }=0. \end{array}$$

Let *G*_*ZZ*_/*G* = *λ*; then we get

(17a) GZZ−λG=0

(17b) R2FRR+RFR+ρω2u02μ+λR2−1F=0.

Equation (17b) is a 1-order Bessel equation, and the solutions are

(18) GZ=A1eλZ+A2e−λZFR=B1J1ρω2u02μ+λR+B2Y1ρω2u02μ+λR,

where* J*_1_,* Y*_1_ are 1-order Bessel function and Neumann function, respectively.

Inputting *U*_*R*_(*Z* → *∞*) = 0, we get *A*_1_ = 0. Because *J*_1_(0) = 0 and *Y*_1_(0) = −*∞*, based on the boundary condition *U*_*R*_(*R* → 0, *Z* = 0) = 0, we get *B*_2_ = 0; therefore

(19) $$\begin{array}{c} U_{R}=AJ_{1}\left(\;\sqrt{\frac{\rho \omega^{2}{u_{0}}^{2}}{\mu }+\lambda }R\;\right)e^{-\sqrt{\lambda }Z}. \end{array}$$

Because *J*_1_(+*∞*) = 0, then *U*_*R*_(*R* → *∞*) = 0.

Put *U*_*Z*_ = *F*(*R*)*G*(*Z*) into (6b),

(20) $$\begin{array}{c} \frac{F_{RR}+\left(1/R\right)F_{R}}{F}+\frac{\rho \omega^{2}{u_{0}}^{2}}{\mu }+\frac{G_{ZZ}}{G}=0. \end{array}$$

Let *G*_*ZZ*_/*G* = *λ*; then we get

(21a) GZZ−λG=0

(21b) FRR+1RFR+ρω2u02μ+λF=0.

Equation (21b) is a 0-order Bessel equation; therefore the solutions are

(22) GZ=A1eλZ+A2e−λZFR=B1J0ρω2u02μ+λR+B2Y0ρω2u02μ+λR.

Inputting *U*_*z*_(*Z* → *∞*) = 0, we get *A*_1_ = 0. Because *J*_0_(0) = 1 and *Y*_1_(0) = −*∞*, based on the boundary condition *U*_*z*_(*R* → 0, *Z* = 0) = 1, we get *B*_2_ = 0; therefore

(23) $$\begin{array}{c} U_{Z}=J_{0}\left(\;\sqrt{\frac{\rho \omega^{2}{u_{0}}^{2}}{\mu }+\lambda }R\;\right)e^{-\sqrt{\lambda }Z}. \end{array}$$

Suppose the tissue is incompressible, that is, $\nabla ∙\vec{v}=0$; therefore

(24) $$\begin{array}{c} \frac{\partial U_{R}}{\partial R}+\frac{U_{R}}{R}+\frac{\partial U_{Z}}{\partial Z}=0. \end{array}$$

Putting (19) and (23) into (24), we get

(25) A∂J1ρω2u02/μ+λR∂R+AJ1ρω2u02/μ+λRR−λJ0ρω2u02μ+λR=0.

Based on the recurrence relation of Bessel's function

(26) Jv−1x+Jv+1x=2vxJvxJv−1x−Jv+1x=2Jv′x,

we get

(27) ∂J1kR∂R=kJ0kR−J2kR2J1kRR=kJ0kR+J2kR2k=ρω2u02μ+λ.

Putting (27) into (25), we get

(28) AkJ0kR−J2kR2+J0kR+J2kR2−λJ0kR=0,

and then $A=\sqrt{\lambda }/k$. Therefore

(29) UZ=J0ρω2u02μ+λRe−λZUR=λρω2u02/μ+λJ1ρω2u02μ+λRe−λZ.

### 2.5. Calculation Solutions of Part (2)

Wang et al. have simplified the tissue as one-dimensional question [15]; therefore (6a) and (6b) is changed to

(30) μ∂2UR∂R2+1R∂UR∂R−URR2=−ρω2u02URμ∂2UZ∂R2+1R∂UZ∂R=−ρω2u02UZ.

Based on $\nabla ∙\vec{v}=0$, the solutions are

(31) UR=0UZ=J0ρω2u02μR.

Both (29) and (31) do not fit very much in this part; therefore, we suppose (31) as the upper boundary condition (*Z* = −*L*_0_/*u*_0_), (29) works as the lower boundary condition (*Z* = 0), linear interpolation is adopted to calculate part (2), that is,

(32) URR,Z=−ZURR,0UzR,Z=UzR,0+ZUzR,0−UzR,−L0u0−L0u0<Z<0.

### 2.6. Parameters

The analytic solution (29) shows that *λ* affects the amplitude attenuation along *z*. Langevin et al.'s research on acupuncture demonstrates the displacement is small when *z* is over 2 cm [10]. Therefore, we suppose the displacement amplitude at *z* = 2 cm is a small amount compared to the amplitude at *z* = 0 cm (the plane of the needle tip), that is $e^{-\sqrt{\lambda }(z/u_{0})}\approx 10\%$; we can determine *λ* ≈ 0.25. The standard *u*_0_ is 0.5 cm and *r*_0_ is 0.02 cm.

The biological viscoelasticity is determined by [16]

(33) $$\begin{array}{c} \mu \left(\;i\omega \;\right)=\frac{1+\left(c/2\right)\left[\ln\left(1+\omega^{2}\tau_{2}^{2}\right)-\ln\left(1+\omega^{2}\tau_{1}^{2}\right)\right]+ic\left[tan^{-1}\left(\omega \tau_{2}\right)-tan^{-1}\left(\omega \tau_{1}\right)\right]}{1+c\ln\left(\tau_{2}/\tau_{1}\right)}. \end{array}$$

where *τ*_1_ = 0.0984, *τ*_2_ = 8454.76, *c* = 0.0351.

$J_{0}(\sqrt{\rho \omega^{2}{u_{0}}^{2}/\mu +\lambda }R)$ and $J_{1}(\sqrt{\rho \omega^{2}{u_{0}}^{2}/\mu +\lambda }R)$ will diverge at *R* → *∞* if *ρω*^2^*u*_0_^2^/*μ* + *λ* have an imaginary part. Obviously, *λ*>0; notice that the imaginary part of *ρω*^2^*u*_0_^2^/*μ* is small compared to the real part; for example, *ρω*^2^*u*_0_^2^/*μ* = 1.2 − 0.03*i* when *ω*=2*π* and *ρω*^2^*u*_0_^2^/*μ*= 76 − 0.38*i* when *ω*=16*π*; therefore we replace $\sqrt{\rho \omega^{2}{u_{0}}^{2}/\mu +\lambda }$ with its module $\left|\sqrt{\rho \omega^{2}{u_{0}}^{2}/\mu +\lambda }\right|$ to void divergence.

## 3. Results

### 3.1. Boundary (*Z* = 0, *Z* = −*L*_0_/*u*_0_) Solutions

**Figure 3:**
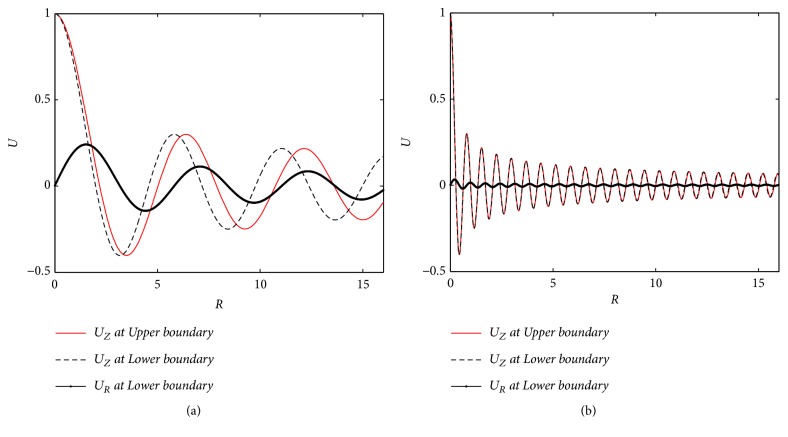
Amplitudes of tissue displacements at the upper (*Z* = −*L*_0_/*u*_0_) and lower (*Z* = 0) boundaries. (a) *ω* = 2*π*; (b) *ω* = 16*π*.

Obviously, the upper (*Z* = −*L*_0_/*u*_0_) and lower (*Z* = 0) boundary solutions determine displacement distributions in the skin tissue. The dimensionless tissue displacements (*U*_Z_ at the upper and lower boundaries and the *U*_R_ at the lower boundary) at two stimuli frequencies (*ω* = 2*π* and *ω* = 16*π*) are plotted in Figure 3. The distributions of *U*_Z_ are very similar at upper and lower boundaries.

**Figure 4:**
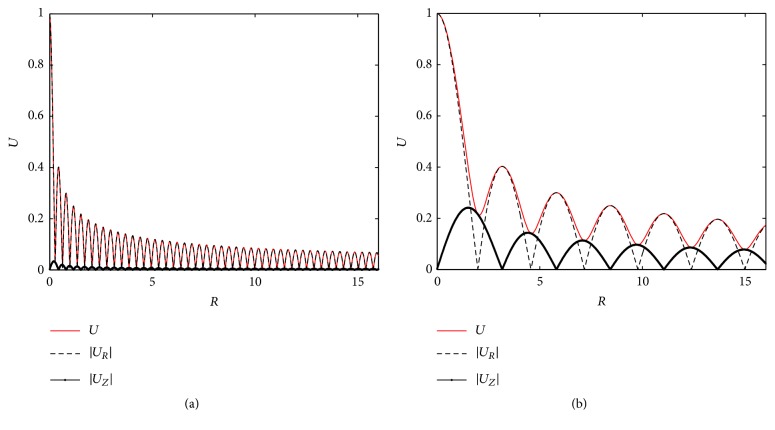
Amplitudes of tissue displacements at *Z* = 0. (a) *ω* = 2*π*; (b) *ω* = 16*π*.

**Figure 5:**
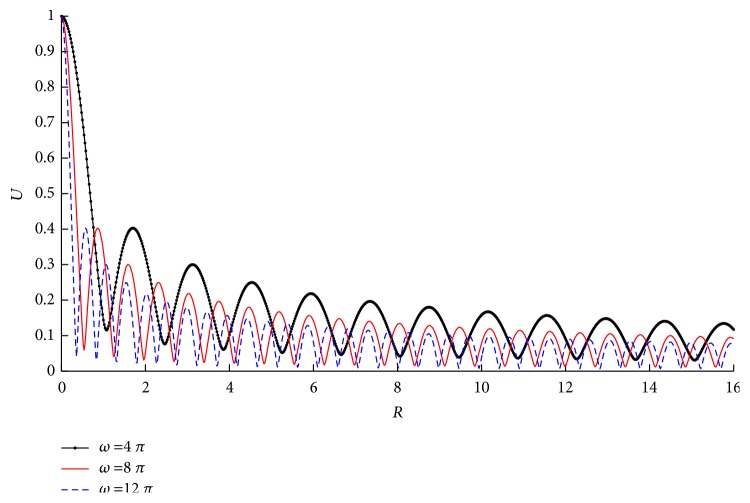
Amplitudes of tissue displacements under different frequencies.

Figure 4 shows the corresponding absolute values of displacements (|*U*_R_| and |*U*_Z_|) at lower boundary (*Z* = 0). Additionally, the combined displacement $U=\sqrt{U_{R}^{2}+U_{Z}^{2}}$ is plotted. It shows that* U* is nearly the same as |*U*_Z_| and |*U*_R_| is relatively small, especially at high stimulus frequency (*ω*=16*π*, in Figure 4(b)). Therefore, we will only study* U* afterwards. The influence of stimulus frequency on the displacement (*U*) is shown in Figure 5. It is clear that* U* decreases with the increase of* R*, and at higher frequencies, it decreases more rapidly.

**Figure 6:**
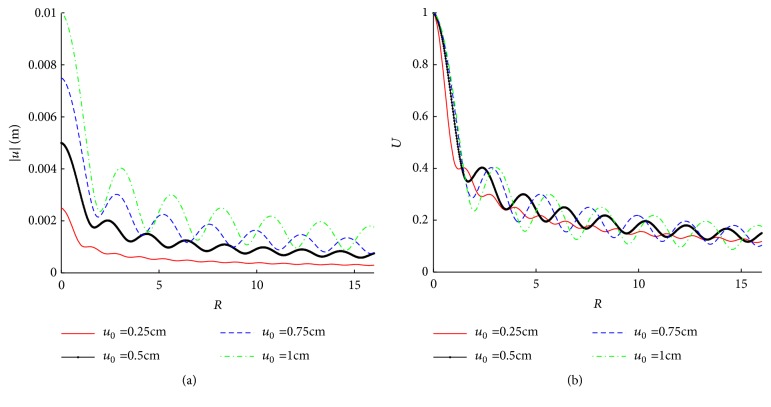
Amplitudes of tissue displacements under different* u*_0_s at *ω* = 2*π*; (a) |*u*|. (b) *U*.

**Figure 7:**
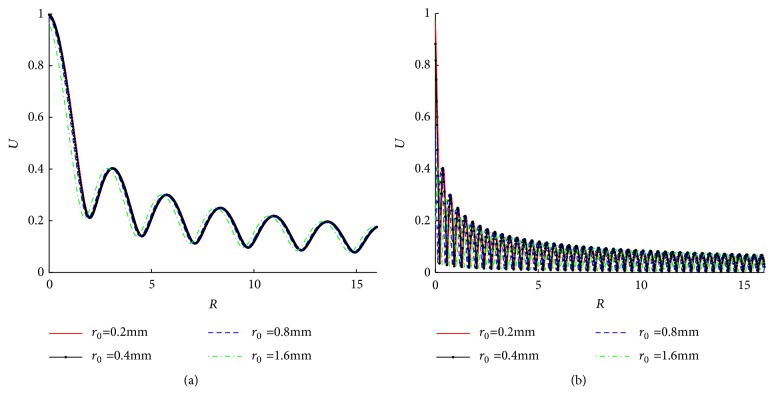
Amplitudes of tissue displacements under different* r*_0_s. (a) *ω* = 2*π*; (b) *ω* = 16*π*.

The influence of needle movement amplitude (*u*_0_) is shown in Figure 6.  Figure 6(a) plots the absolute values of tissue displacement (|*u*|) at the lower boundary with different *u*_0_s (0.25, 0.5, 0.75, and 1.0cm). The range of |*u*| is largely dependent on *u*_0_, with a nearly linear relationship at *R* → 0.  Figure 6(b) shows the dimensionless displacement *U* = |*u*|/*u*_0_, which attenuates a little more slowly along* R* direction with an increasing *u*_0_.  Figure 7 demonstrates *U* in the cases of different needle radius (*r*_0_). It shows that* r*_0_ has a minimal effect on* U*.

### 3.2. Displacement Amplitude (U) Distribution in the Skin Tissue

**Figure 8:**
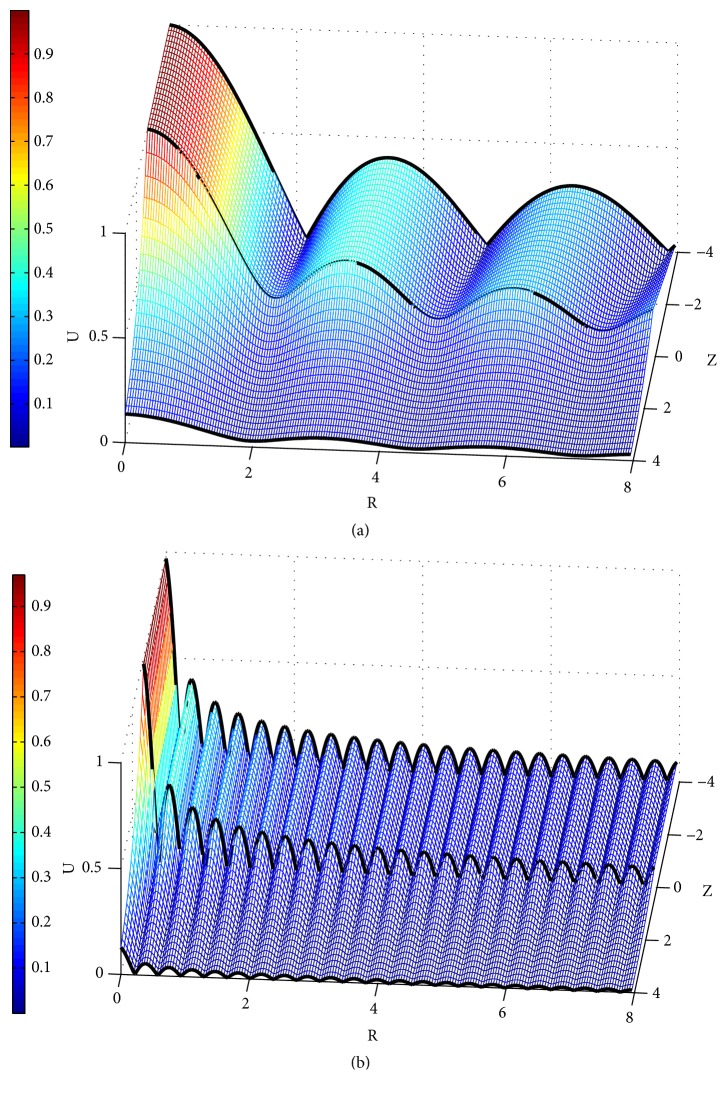
Amplitudes of tissue displacements; the black lines represent the displacement at *Z* = −*L*_0_/*u*_0_ = −4, *Z* = 0 and *Z* = *L*_0_/*u*_0_ = 4. (a) *ω* = 2*π*; (b) *ω* = 16*π*.

Figure 8 shows the displacement amplitude* U* distribution at *ω* = 2*π* and *ω* = 16*π*. Comparing Figures 8(a) and 8(b) shows that the tissue displacement around the needle (−*L*_0_/*u*_0_ ≤ *Z* ≤ 0) decays along the radial direction* R* under both low and high frequency stimulus, but* U* decays less rapidly under low frequency (*U* ≤ 50% where* r* > 0.6 cm) than that under high frequency (*U* ≤ 50% where* r* > 0.07 cm). Moreover,* U* also decays quickly under the needle tip (*Z* > 0).

### 3.3. Shear Strains (*ε*_*rz*_) and Stresses (*τ*_*rz*_) under Different Frequencies

**Figure 9:**
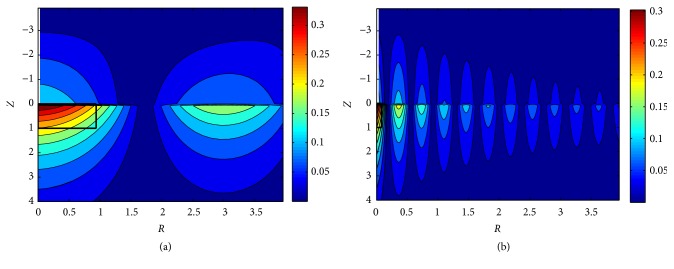
Contour lines of |*E*_*RZ*_| in the tissue; the white lines at *r* → 0∩−4 ≤ *Z* ≤ 0 represent the needle; the black rectangles represent the approximate area where |*E*_*RZ*_| > 0.2. (a)* ω* = 2*π*; (b)* ω* = 16*π*.

**Figure 10:**
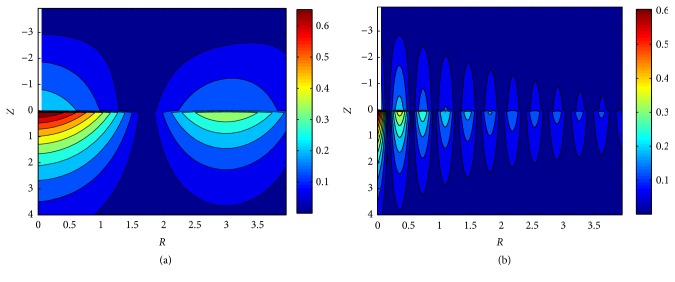
Contour lines of |*T*_*RZ*_| in the tissue, the white lines at *R* → 0∩−4 ≤ *Z* ≤ 0 represent the needle. (a)* ω* = 2*π*; (b)* ω* = 16*π*.

Figure 9 shows the contour lines of shear strain amplitude in absolute values (|*E*_*RZ*_|) in the tissue. The needle is located at* R *= 0, −4 ≤ *Z* ≤ 0 (shown as white lines in Figure 9). The maximum shear strain occurs at the needle tip, and again, strains decay along the radial direction, with more rapid attenuation under high frequency stimulus. Thus, the low frequency stimulus affects larger area than high frequency stimulus. Take the area where |*E*_*RZ*_|> 0.2 as the effective influence region *S*_eff_, and define* Z* and* R* at |*E*_*RZ*_| ≈ 0.2 as *Z*_thr_ and *R*_thr_, respectively; then *S*_eff_ =*Z*_thr_×*R*_thr_≈1×0.94 at* ω* = 2*π* and *S*_eff_ ≈ 1×0.08 at* ω* = 16*π*, shown as the black rectangles in Figure 9. It turns out that the stimulus frequency has little effect on *Z*_thr_ but has significant influence on *R*_thr_.  Figure 10 shows the contour lines of dimensionless shear stress amplitude in absolute values (|*T*_*RZ*_|); it shows the same tendency as |*E*_*RZ*_|.

**Figure 11:**
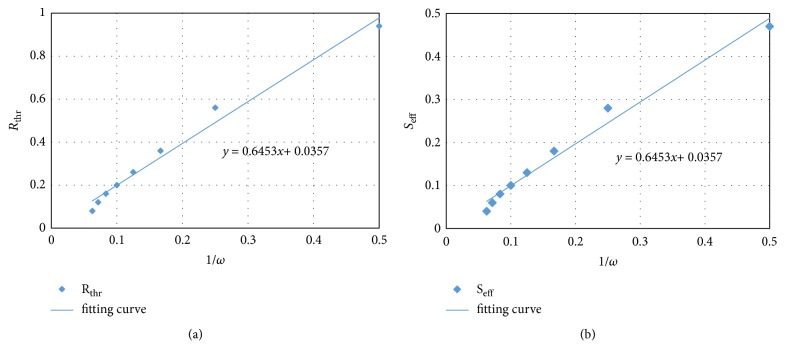
Relationship between *R*_thr_s and *S*_eff_s on one side and 1/*ω*s on the other. Diamonds represent calculating results and blue lines represent the fitting curve.

Table 1 lists *R*_thr_s and *S*_eff_s under different frequencies.  Figure 11 shows that there is an approximately linear relationship between *R*_thr_s and the reciprocal of the stimulus frequency 1/*ωs*. The same relationship also exists between *S*_eff_s and 1/*ω*s.

### 3.4. Energy Dissipation (Δ*E*) and Mechanical Quality Factor (*Q*) under Different Frequencies

**Figure 12:**
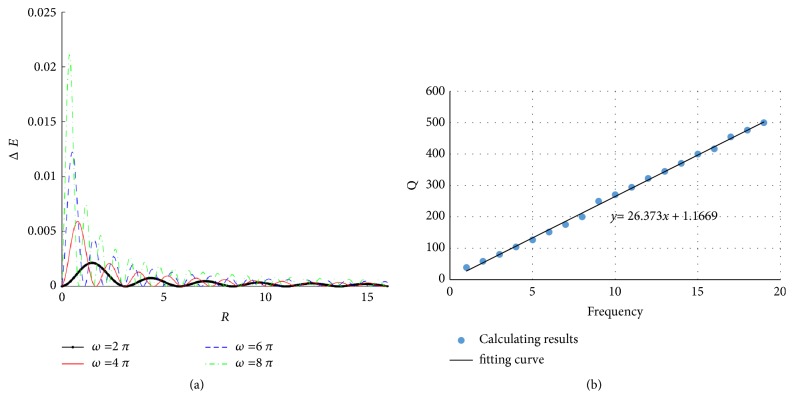
Δ*E* and* Q* under different frequencies. (a) Δ*E* at *Z* = 0; (b) *Q*.

Figure 12 shows Δ*E* and* Q* under different frequencies.  Figure 12(a) shows that Δ*E* is larger and more intensified around the needle at high frequencies than that at low frequencies. For the mechanical quality factor* Q*, a nearly linear relationship between *Q*s and the stimulus frequencies can be found (shown in Figure 12(b)). This means that energy storage ratio increases with the increase of stimulus frequency, and the tissue demonstrates more “elastic” at high frequencies.

## 4. Discussion

Langevin et al. claimed that acupuncture manipulation would lead to tissue displacement and generate a mechanical stress field [10]. This phenomenon is well demonstrated in our simulation. Moreover, the local displacement, strain, stress, and energy near the acupoint (or the needle) have been quantitively analyzed. The characteristics of the tissue displacement are concluded as (1) it is larger at low frequencies (see Figure 8) given other conditions the same (the needle movement* u*_0_ and the material properties, etc.), and (2), the displacement will decay more slowly and extend relatively further from the needle at low frequencies (see Figure 5).

**Figure 13:**
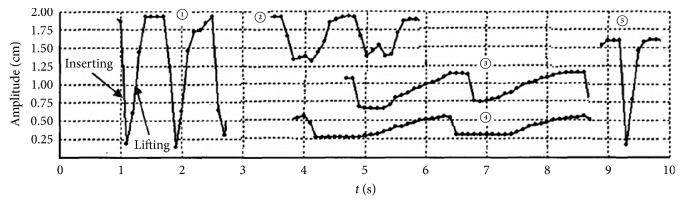
Characteristic curves of five professional acupuncturists.

Main characteristics in the mechanical process of acupuncture are the stimulus amplitude (*u*_0_) and the angular frequency (*ω*). However, there is a lack of consistency of these characteristics. Wang XM pointed out that mechanical information can only influence nearer region of the acupoint [17]. According to our simulation, the resultant tissue displacement is nearly proportional to* u*_0_ (see Figure 6(a)). It is also shown that the displacement, strain, and stress under low frequency stimulus decay more slowly than those under high frequency (see Figures 8–10). These results indicate that the mechanical information (displacement, strain, and stress) can propagate further under low frequency stimulus.  Figure 13 shows the characteristic curves of different acupuncturists, where significant differences in *u*_0_ and *ω* can be found, but *u*_0_ is larger at high frequencies; thus *u*_0_/*ω* tends to be constant [18].  Figure 11(a) shows that there is nearly an inverse relationship between *R*_thr_s and* ω*s, or *R*_*thr*_ = *k*/*ω*, where *k* is a constant. Given *R* = *r*/*u*_0_, then the radius of the effective influence area *r*_*thr*_ = *u*_0_ × *R*_*thr*_ = *u*_0_ × *k*/*ω* = *k*(*u*_0_/*ω*). Thus a constant *u*_0_/*ω* is equivalent to a constant *r*_*thr*_; therefore, to get good acupuncture effect, a sufficient area of tissue should be deformed, which may be an alternative interpolation of acupuncture sensations in the mechanical viewpoint. However, our model is not able to figure out what “sufficient area” should be, which requires further experimental research. But suggestions on achieving mechanical sensations can be made. Since needle movement is a damage process to the tissue and slip between the needle movement and tissue movement may occur at large *u*_0_ (we suppose there is no slip because of small *u*_0_), thus *u*_0_ is limited. Therefore, to achieve better effect (larger effective influence region), low frequency stimulus is a good choice. It was reported that the frequency of acupuncture sensations is concentrated at low frequency [18]. Ding et al. reported the mean frequency to achieve acupuncture sensations by experienced acupuncturists is 1.2 Hz [19].

Figure 7 shows that needle diameter has no effect on displacement. Li et al. reported needling of different diameters induced the consistent change of fascial connective tissues and collagenous fiber arrangement [20]. Figures 9 and 10 show that strain and stress decay quickly under the needle tip. That means the needle must be inserted into the right depth (about 2 cm in lower limbs) and position (*π*/*ω* cm around the acupoint in our simulation) to induce enough strain and stress. A survey showed that acupuncturists think deep insertion was easier to induce acupuncture sensations than shallow insertion [21].

Because of the limitation of experimental means, it is impossible to measure tissue strain and stress in vivo, tissue displacement, or the stress on the needle are exploited to infer the tissue strain [22]. But there is a complicated relationship between strain and displacement; it is controversial to infer stress field without considering needle frequency. Our model predicted the relationship between the main deformation areas and frequencies (see Figure 11). But there is no agreement on the threshold of |*E*_*RZ*_| to achieve acupuncture effect and tissue is more complicated (anisotropy, nonlinearity) than our model [23]; further experiments are required to testify the results.

Figure 12(a) shows that energy dissipation is relatively small under low frequency compared with that under high frequency; Figure 10(b) shows that there is a linear relationship between mechanical quality factors and frequencies. Obviously, a high energy is required to maintain a high frequency manipulation.

## 5. Conclusion 

In this study, we have illustrated that lifting-thrusting needle will lead to tissue displacements and generate a mechanical stress field. The displacement correlated with needle frequency and decayed more slowly under low frequency than that under high frequency. Strain and stress correlated with frequency too, and there exists an inverse relationship between the main deformation areas and frequencies. Energy dissipation is also affected by frequency. This is a preliminary study of mechanical effects of acupuncture manipulation, isotropy, and quasi-linear viscoelastic theory adopted to simplify soft tissue, while Fox et al. observed anisotropic tissue motion induced by acupuncture [24]. Our results may reflect local effects around the needle; anisotropy must be considered if we study the mechanical information propagation in the tissue.

## Figures and Tables

**Table 1:** *R*_thr_s and *S*_eff_s under different angular frequencies.

*ω*	2*π*	4*π*	6*π*	8*π*	10*π*	12*π*	14*π*	16*π*
*R*_thr_	0.94	0.56	0.36	0.26	0.20	0.16	0.12	0.08

*S*_eff_	0.5×0.94	0.5×0.56	0.5×0.36	0.5×0.26	0.5×0.20	0.5×0.16	0.5×0.12	0.5×0.08

## Data Availability

The data used to support the findings of this study are included within the article. We declared that materials described in the manuscript, including all relevant raw data, will be freely available to any scientist wishing to use them for noncommercial purposes, without breaching participant confidentiality.
